# DERL3 facilitates the progression of clear cell renal cell carcinoma by promoting epithelial-mesenchymal transition via regulation of the TGFB1 pathway

**DOI:** 10.1371/journal.pone.0322172

**Published:** 2025-04-29

**Authors:** Chengtao Niu, Haodong Cui, Xintong Sun, Yuchang Yang, Xue Jiang, Zixiang Cong, Yiming Zhang, Zhihong Niu, Wei He

**Affiliations:** 1 Department of Urology, Shandong Provincial Hospital, Shandong University, Jinan, Shandong, China; 2 Department of Urology, Shandong Provincial Hospital Affiliated to Shandong First Medical University, Jinan, China; 3 Shandong Xiehe University, Jinan, China; 4 Department of Urology, Weihai Municipal Hospital Affiliated to Shandong University, Weihai, China; 5 Department of Urology, People’s Hospital of Changle County, Weifang, Shandong, China; The University of British Columbia Life Sciences Institute, CANADA

## Abstract

**Background:**

Clear cell renal cell carcinoma (ccRCC) is a highly prevalent malignancy within the urinary system. The intrinsic heterogeneity and resistance to conventional chemotherapy and radiotherapy contribute to the poor prognosis of advanced ccRCC patients. DERL3, part of the Derlin protein family, was first identified for its critical role in endoplasmic reticulum stress. Subsequent studies have revealed its involvement in the progression of multiple tumor types; however, its role in ccRCC remains unclear.

**Methods:**

In this study, we utilized bioinformatics analysis and in vitro experimental approaches to investigate the role of DERL3 expression in the metastasis of renal clear cell carcinoma cells. Additionally, we analyzed the correlation between DERL3 expression and the prognosis of renal clear cell carcinoma patients, while exploring its potential mechanisms of action.

**Results:**

We demonstrate for the first time that DERL3 promotes tumor progression in ccRCC, showing significantly elevated expression, especially in metastatic ccRCC cell lines. Further studies suggest that this overexpression of DERL3 may promote the epithelial-mesenchymal transition in ccRCC by upregulating TGF-β1, thereby enhancing ccRCC metastasis.

**Conclusion:**

In conclusion, our study clarifies the role and potential mechanisms of DERL3 in ccRCC progression, providing promising therapeutic avenues for improving the prognosis of ccRCC patients.

## Introduction

Clear cell renal cell carcinoma (ccRCC) is a disease with an increasing global incidence, with an estimated 400,000 new cases and approximately 175,000 deaths from kidney cancer each year. The expansion of routine imaging investigations for various diseases has led to an increasing number of patients with ccRCC being diagnosed incidentally [1–3]. Despite advancements in the diagnosis and treatment of ccRCC over the past two decades, it remains one of the most lethal malignancies of the urinary system [4]. Among RCC subtypes, ccRCC is the most prevalent histological variant [5]. Surgical intervention is currently the preferred treatment for localized ccRCC, which generally has a favorable prognosis [6]. However, for patients with advanced ccRCC characterized by surrounding tissue invasion and distant metastasis, surgical outcomes are suboptimal, and the efficacy of radiotherapy and chemotherapy is limited [7]. The application of targeted therapies and immunotherapies has, to some extent, stabilized disease progression and prolonged patient survival [8,9]. Identifying targetable molecular markers that drive the progression of ccRCC is imperative.

DERL3, a member of the Derlin family, is located on chromosome 22q11.23. Its mature protein, Derlin-3, is primarily localized to the endoplasmic reticulum (ER) membrane and shows homology to Der1p (a transmembrane protein involved in ER-associated degradation in yeasts) [10]. Initially described as participating in the protein degradation processes associated with ER stress, Derlin-3 may play a role in the formation of ER-associated degradation complexes [10–12]. Endoplasmic reticulum stress (ERS) is caused by various factors such as oxidative stress and chemical injury, resulting in physiological dysfunction of the ER and the accumulation of misfolded or unassembled proteins. ERS plays a significant pathological role in numerous diseases, including metabolic disorders, cancer, and neurological diseases [6]. Relevant studies have indicated that ERS is involved in the progression of ccRCC [13,14]. As a key factor in the ER stress response, existing research suggests that the overexpression of DERL3 is associated with aggressive phenotypes in breast cancer cells and is implicated in the occurrence and progression of lung adenocarcinoma [12,15]. Other studies indicate that DERL3 may exert an inhibitory effect in colorectal cancer, while the epigenetic loss of DERL3 induces the overexpression of SLC2A1, thereby influencing tumor progression through the Warburg effect [16,17]. However, the role of DERL3 in the progression of ccRCC and its underlying mechanisms remain unreported.

The transdifferentiation of epithelial cells into motile mesenchymal cells is referred to as epithelial-mesenchymal transition (EMT) [18]. This process is a critical component of development, wound healing, and stem cell behavior, and it pathologically contributes to fibrosis and cancer progression [19]. Recent studies have indicated that the EMT in ccRCC may facilitate the progression of this disease [20]. However, the intricate mechanisms underlying the role of EMT in ccRCC progression remain unclear [21]. Therefore, investigating the significance of DERL3 in the pathogenesis of ccRCC may aid in identifying new therapeutic targets for this cancer.

In this study, our team provides the first key evidence that DERL3 promotes the progression of ccRCC. We observed that DERL3 is highly expressed in ccRCC, particularly in metastatic ccRCC cell lines. Further mechanistic studies revealed a positive correlation between DERL3 expression and the expression of TGFB1 and stromal cell-related proteins, suggesting that high DERL3 expression enhances ccRCC progression by promoting EMT.

In summary, our research further confirms the crucial role of DERL3 in ccRCC metastasis, elucidates the underlying molecular mechanisms of ccRCC progression, and emphasizes the potential of DERL3 as a novel therapeutic target for this disease.

## Materials and methods

### Clinical specimens

This study involves the collection of nine surgical specimens from patients diagnosed with renal ccRCC at Shandong First Medical University Affiliated Provincial Hospital on August 21, 2024. The specimens were fixed with formalin and embedded in paraffin. None of the patients had received neoadjuvant therapy prior to surgery. Two experienced pathologists confirmed the diagnosis of ccRCC for all specimens. This study did not involve minors. The specimens collected were routine pathological specimens sent to the pathology department after surgery, and the patients had their informed consent and signed the informed consent form. This study protocol conformed to the ethical guidelines of the 1975 Declaration of Helsinki and was approved by the Medical Ethics Committee of Shandong Provincial Hospital (No. SWYX2024–431).

### Data sources and databases

RNA-seq data from ccRCC samples, along with corresponding clinical and follow-up data, were retrieved from TCGA portal (https://portal.gdc.cancer.gov/). The RNA-seq data were structured into an expression matrix for further analysis. The GEO query package in R facilitated access to the Gene Expression Omnibus (GEO) database (https://www.ncbi.nlm.nih.gov/geo/) for downloading the GSE40435 dataset, thereby validating the expression analysis results from the TCGA database.

### Pan-cancer expression analysis and Kaplan-Meier analysis

The pan-cancer expression analysis of DERL3 was conducted using the TIMER 2.0 database. Kaplan-Meier curve analysis was performed via the GEPIA database, integrating data from TCGA and the Genotype-Tissue Expression GTEx project, to evaluate the impact of DERL3 expression on overall survival (OS) and disease-free survival (DFS).

### Gene set enrichment analysis (GSEA)

We conducted gene set enrichment analysis using the GSEA_4.3.3 software. Subsequently, enrichment plots were generated to further evaluate the GSEA results. A nominal p-value of less than 0.05 and a false discovery rate FDR q-value of less than 0.25 were considered statistically significant.

### Cell culture and reagents

The human renal tubule epithelial cell line HK-2 and several ccRCC cell lines (ACHN, Caki−1, 786−O, and A498) were acquired from the Shanghai Cell Bank of the Chinese Academy of Sciences. HK-2 cells were cultured in DMEM-Ham’s F12 medium (MACGENE, China) supplemented with 10% fetal bovine serum (EXcellBio, China) and 1% penicillin-streptomycin (Solarbio, Beijing). Caki-1 cells were maintained in McCoy’s 5A medium (MACGENE, China) under similar conditions. ACHN cells were cultured in MEM (MACGENE, China) while A498 and 786-O cells were cultured in DMEM and RPMI-1640 mediums (MACGENE, China) respectively, with the same supplemental components.

### Cell lentivirus infection experiment

The cells in the logarithmic growth phase were digested with trypsin and centrifuged to prepare a cell suspension, which was counted and plated in a 6-well plate and cultured overnight. When the cells grew to 40%-50% confluence, the corresponding volume of lentivirus suspension was added according to different infection multiplicity (MOI = 5, 10, 15, 30) for infection. After incubation for 24 hours, the culture medium was replaced and cultured. After 3 days of infection, when the cells reached 100% confluence, the infection efficiency of each well was observed under a fluorescence microscope, and the optimal MOI value was determined to be 15. According to the optimal MOI value determined in the preliminary experiment, the cells were infected, the calculated volume of virus stock solution was added, and fresh culture medium was replaced after incubation at 37°C for 24 hours, and the culture was continued for 48–72 hours to complete the infection.

The sequence information used in this experiment is as follows:

NC: 5’TTCTCCGAACGTGTCACGT3’
sh-DERL3–1: 5’GTGGGCCATATCTACTACTTC3’;
sh-DERL3–2: 5’CGACTTCGTCTTCATGTTTCT3’;
sh-DERL3–3: 5’CCTGTCCATGTTGTGGTTTAT3’;
sh-TGFβ1–1: 5’CCCGCGTGCTAATGGTGGAAA3’;
sh-TGFβ1–2: 5’CCGGCCTTTCCTGCTTCTCAT3’;
sh-TGFβ1–3: 5’CGCCTGTAATCCTAGCACTTT3’;

### Reverse transcription quantitative PCR (RT-qPCR)

RNA was extracted from ccRCC and adjacent tissue using the TRNzol Universal Total RNA Extraction Kit (https://www.tiangen.com/asset/imsupload/up0906252001604560423.pdf). cDNA synthesis was performed in accordance with the FastQuant cDNA First-Strand Synthesis Kit KR116 protocol (https://www.tiangen.com/asset/imsupload/up0265627001604562652.pdf). Specific primers were designed based on the target gene sequences, and SYBR Green fluorescent quantitative PCR reagents (SuperReal PreMix, FP205) were employed for analysis, utilizing cDNA as the template. Each sample was analyzed in triplicate, and the experimental data were evaluated using the 2-ΔΔCT method.

### SDS-PAGE and Western blotting

To extract and quantify proteins, cells were lysed using Western and IP cell lysis buffer (P0013J, Beyotime Biotechnology, Shanghai), with PMSF (ST506, Beyotime Biotechnology, Shanghai) added to inhibit protease activity. Protein concentration was determined using the BCA method, following the manufacturer’s instructions. Signal detection was performed using the Tanon-4600 chemiluminescence imaging system (Beijing Yuanpinghao Biotechnology Co., Ltd.), with all steps following the manufacturer’s recommendations and standard operating procedures. In the experimental results, each band was clearly identified.

Primary Antibody (Dilution ratio): GAPDH (1:1000), DERL3 (1:1000), TGF-β1 (1:1000), E-cadherin (1:1000), Vimentin (1:1000), α-SMA (1:1000), Fibronectin (1:1000).

Secondary Antibody (Dilution ratio): Goat Anti-Mouse (1:2000), Goat Anti-Rabbit (1:2000).

### MTT assay for cell proliferation

The cells in the logarithmic growth phase were suspended and treated with MTT solution for four hours. Subsequently, dimethyl sulfoxide DMSO (CM15019, MACGENE) was added, and the absorbance at 490 nm was measured using an ELISA reader.

### Colony formation assay

Cells in the logarithmic growth phase were digested with trypsin, centrifuged, and subsequently counted after resuspension. The cells were then inoculated into 6 cm culture dishes and cultured for a duration of 10 days. Following fixation, staining, and washing, the colonies were photographed and quantified using ImageJ software.

### Migration assay

The digested cells were prepared into a single cell suspension and the concentration was adjusted to 2.5×10⁵/ml. Transwell chambers were assembled in 24-well plates, 500 μl of medium containing 10% FBS was added to the lower chamber, and 300 μl of serum-free medium containing cells was added to the upper chamber. After the chamber was incubated at 37°C for 24 hours, it was rinsed with PBS, fixed with 4% paraformaldehyde for 30 minutes, and rinsed with PBS three times. Subsequently, it was stained with 0.1% crystal violet stain for 10 minutes, rinsed with running water to remove non-migrated cells, and dried naturally. Three fields of view (100×, 200×) were randomly selected under a fluorescence microscope to capture images, count, and calculate the average value.

### Invasion assay

The Matrigel matrix was melted, diluted and coated on the upper chamber of the Transwell chamber, 60–100 μl per well. After digesting the cells, a single cell suspension was prepared and the concentration was adjusted to 2.5×10⁵/ml. The Transwell chamber was assembled in a 24-well plate, 500 μl of culture medium containing 10% FBS was added to the lower chamber, and 300 μl of serum-free culture medium containing cells was added to the upper chamber. After incubating the chamber at 37°C for 24 hours, it was rinsed with PBS, fixed with 4% paraformaldehyde for 30 minutes, and stained with 0.1% crystal violet for 10 minutes. After removing the non-invaded cells, it was naturally dried. Finally, 3 fields of view were randomly selected under a fluorescence microscope to take images, count and calculate the average value.

### Phase contrast microscopy

The proteins in the logarithmic growth phase were digested with trypsin and then washed with PBS to remove impurities. The morphological characteristics of the cells were observed under a phase contrast microscope and images were collected using a computer.

### Statistical analysis

All data are presented as means ± standard deviations. Each experiment was replicated three times. Statistical analyses were conducted using R software version 4.2.2 and GraphPad Prism version 9.0 (La Jolla, CA, USA). Certain statistical data were sourced from online databases. The Shapiro-Wilk test was used for normality test, the Levene test was used for homogeneity of variance test, the two-tailed independent sample t test was used to compare the differences between the two groups, the Pearson correlation coefficient was used for correlation analysis between variables, and the Kaplan-Meier survival analysis was used to evaluate the survival probability between different groups. A p-value of less than 0.05 was considered statistically significant (*p < 0.05, **p < 0.01, and ***p < 0.001).

## Results

### DERL3 is expressed at elevated levels in tissues affected by ccRCC

**Fig 1:**
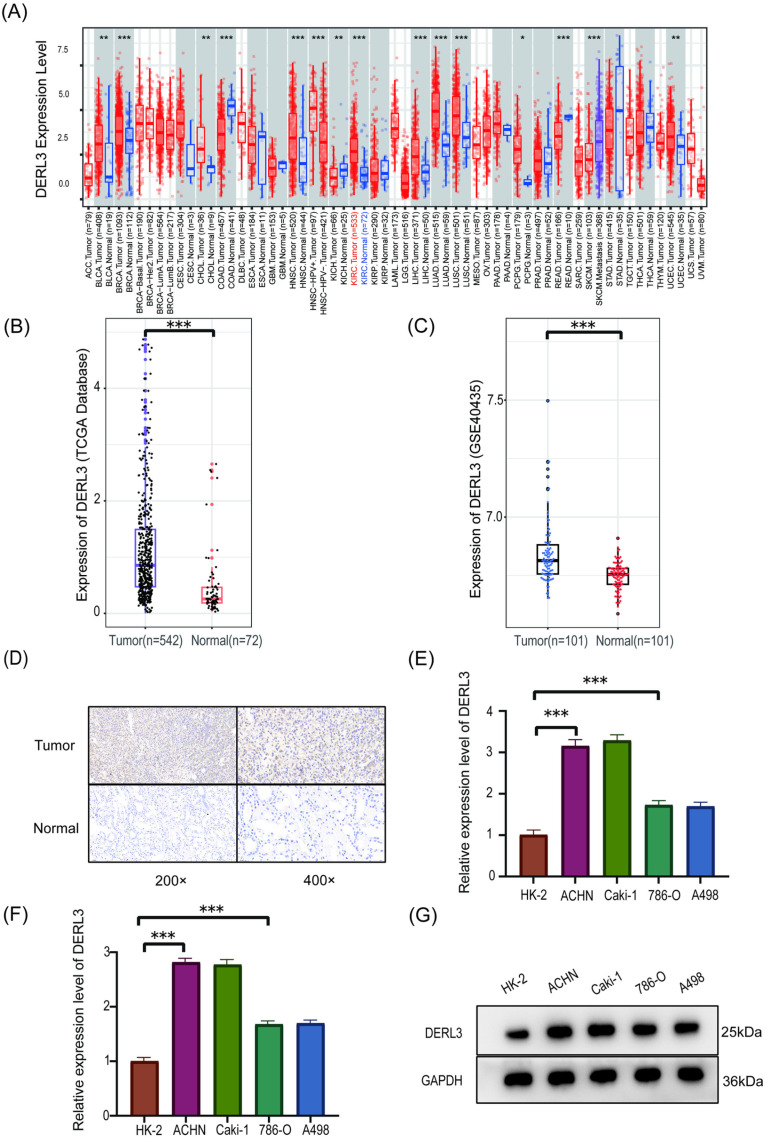
Expression of DERL3 in Pan-Cancer and its expression in ccRCC and adjacent normal tissues. (A): Expression of DERL3 in Pan-Cancer; (B): The relationship between DERL3 gene expression and Group (TCGA); (C): The expression levels of DERL3 in 101 pairs of normal tissues and adjacent cancerous tissues based on the GEO database GSE40435 dataset; (D): Immunohistochemical staining to verify the expression levels of DERL3 in ccRCC; (E): qRT-PCR to verify the expression levels of DERL3 in ccRCC cell lines; (F, G): Western Blot to verify the expression levels of DERL3 in ccRCC cell lines; (***p<0.001).

The DERL3 pan-cancer expression matrix was obtained from the TIMER 2.0 website (http://timer.cistrome.org/). It was observed that the expression of DERL3 is significantly higher in malignant tumors, including clear cell renal carcinoma, bladder cancer, breast cancer, cholangiocarcinoma, hepatocellular carcinoma, lung adenocarcinoma, lung squamous cell carcinoma, thyroid cancer, and endometrial cancer, compared to adjacent normal tissues (Fig 1A). Using R language and the ggplot2 package, we illustrated the expression differences of the DERL3 gene across 72 normal tissue samples and 532 ccRCC tumor tissue samples and collected clinical data from patients for further analysis of clinical relevance. The findings indicated that the expression level of DERL3 in ccRCC is significantly elevated compared to adjacent normal tissues (Fig 1B). An analysis of the GSE40435 dataset confirmed the previously mentioned high expression of DERL3 in ccRCC, with statistically significant results (Fig 1C).

To validate the analysis of public data, we conducted immunohistochemical staining on ccRCC specimens and their adjacent normal tissues obtained from surgery (Fig 1D). Furthermore, qRT-PCR was employed to measure the expression of DERL3 in HK-2, ACHN, Caki-1, 786-O, and A498 cell lines. The results demonstrated that the expression of DERL3 was significantly higher in the ACHN, Caki-1, 786-O, and A498 cell lines compared to the HK-2 group, with ACHN and Caki-1 showing the most pronounced increases (Fig 1E). Additionally, our Western blot analyses revealed a marked elevation in DERL3 expression levels in ccRCC cell lines (Fig 1F, G).

In summary, the expression of DERL3 in ccRCC is significantly higher than that in surrounding normal tissues.

### High expression levels of DERL3 are correlated with unfavorable clinicopathological characteristics

Based on the validation mentioned above, DERL3 is highly expressed in ccRCC tumor tissues. We further explored the association between DERL3 expression levels and the clinicopathological characteristics of ccRCC patients.

**Fig 2:**
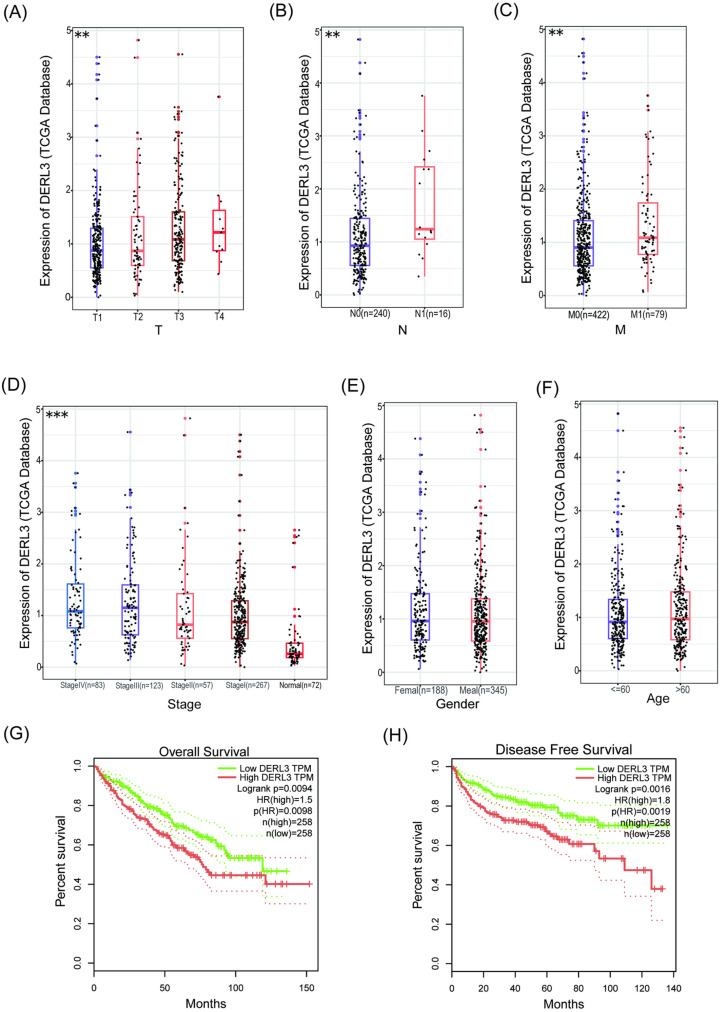
Correlation of DERL3 expression with clinicopathological features and prognosis of ccRCC. The relationship between DERL3 gene expression and T stage (A), N stage (B), M stage (C), Clinical Stage (D) and Gender (E), Age (F) of ccRCC; (G): Kaplan-Meier survival curve for Overall Survival in patients with high and low DERL3 expression; (H): Kaplan-Meier survival curve for Disease-Free Survival in patients with high and low DERL3 expression; (**p<0.01, ***p<0.001).

We analyzed 532 ccRCC tissue samples from the TCGA database. The results showed that high DERL3 expression was significantly correlated with advanced T stage (Fig 2A), lymph node infiltration (N) (Fig 2B), distant metastasis (M) (Fig 2C), and clinical stage (Fig 2D). However, no significant correlation was found with patient gender (Fig 2E) or age (Fig 2F). Further Kaplan-Meier analysis revealed that the high DERL3 expression group had shorter OS (Fig 2G) and DFS (Fig 2H) compared to the low expression group. These results suggest that high DERL3 expression is associated with poor prognosis in ccRCC.

### The verification of DERL3 overexpression and knockdown efficiency and TGF-β1 knockdown efficiency

**Fig 3:**
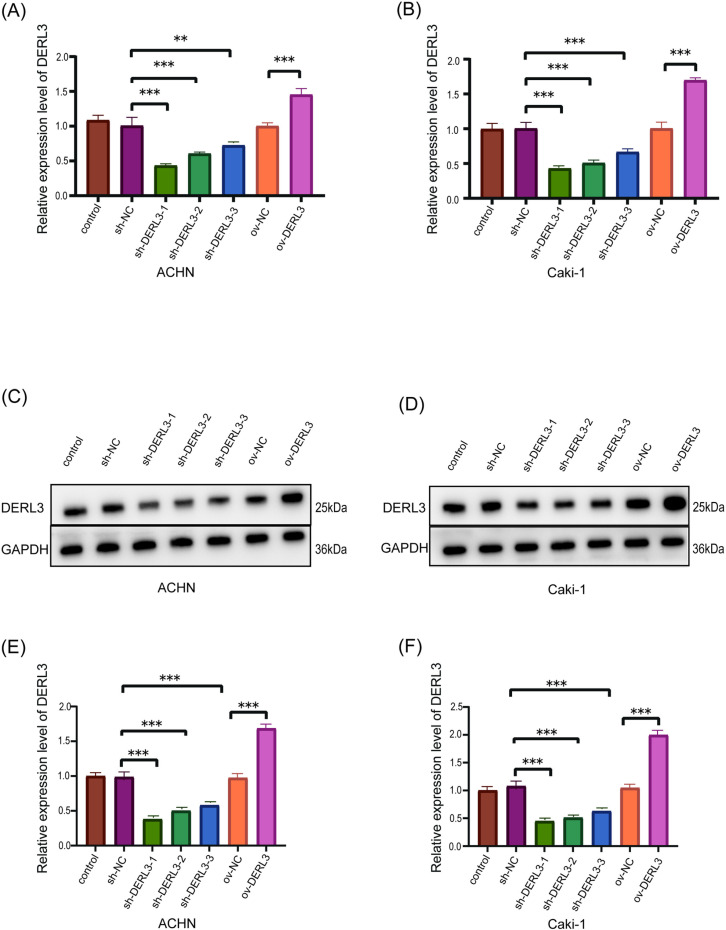
Verification of DERL3 overexpression and knockdown efficiency. (A, B): mRNA expression levels of DERL3 after overexpression and knockdown of DERL3 in ACHN and Caki-1 cell lines; (C, D, E, F): Protein expression levels after DERL3 overexpression and knockdown in ACHN and Caki-1 cell lines; (**p<0.01, ***p<0.001).

We established DERL3 overexpression and knockdown models using ACHN and Caki-1 cell lines. The expression levels of DERL3 following overexpression and knockdown were assessed through qRT−PCR and Western blot analysis. The qRT-PCR results indicated no significant differences among the ov-NC, sh-NC, and control groups. In contrast, the overexpression group (ov−DERL3) exhibited a significant increase in DERL3 expression compared to the ov-NC group. Additionally, the knockdown groups sh−DERL3−1, sh−DERL3−2, sh−DERL3−3 showed a marked reduction in DERL3 expression compared to the sh-NC group, with sh-DERL3–1 displaying the highest knockdown efficiency. These findings confirm the effectiveness of both DERL3 overexpression and knockdown (Fig 3A, B). Similarly, the Western blot results further validated the successful overexpression and knockdown of DERL3 (Fig 3C–F).

**Fig 4:**
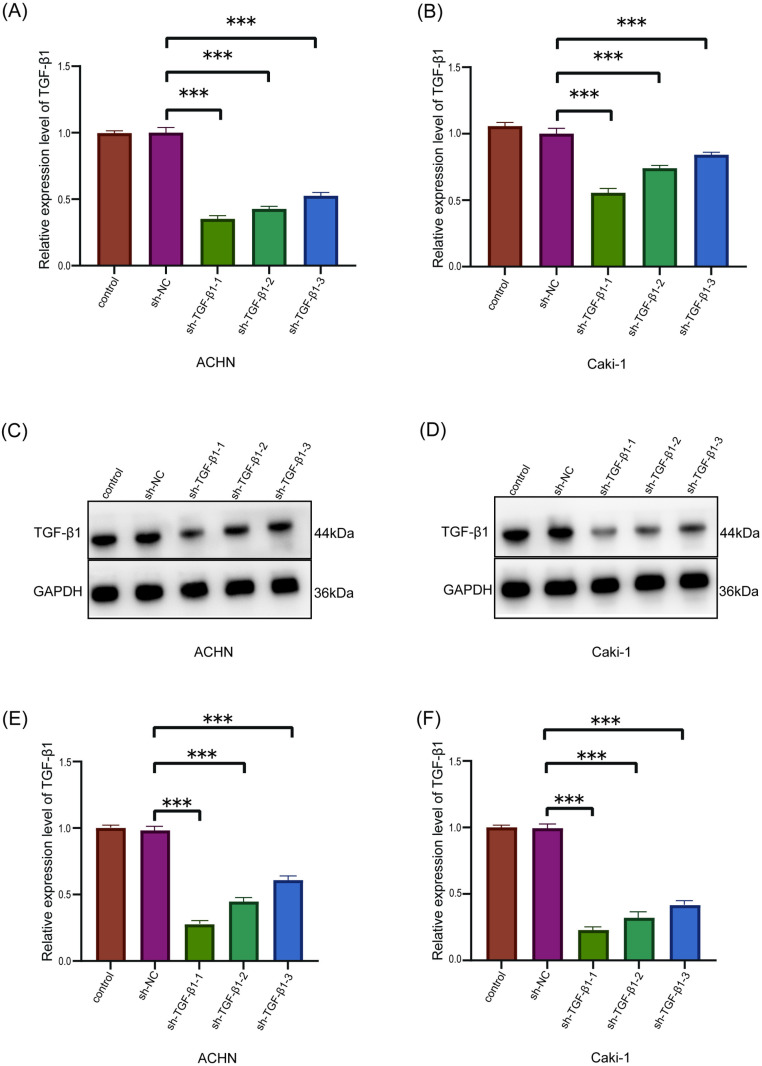
TGF-β1 knockdown efficiency verification. (A, B): mRNA expression level of TGF-β1 after knockdown of TGF-β1 in ACHN and Caki-1 cell lines; (C, D, E, F): protein expression level after knockdown of TGF-β1 in ACHN and Caki-1 cell lines; (***p<0.001).

In the TGF-β1 knockdown model, the expression of TGF-β1 in the knockdown group sh-TGF-β1–1, sh-TGF-β1–2, and sh-TGF-β1–3 was significantly reduced compared with the sh-NC group., among which the sh-TGF-β1–1 group has the highest knockdown efficiency. We will continue to use sh-TGF-β1–1 for subsequent experiments. These findings confirmed the effectiveness of TGF-β1 knockdown. Likewise, Western blotting results further verified the success of TGF-β1 knockdown (Fig 4).

### DERL3 overexpression promotes the proliferation, migration, and invasiveness of ccRCC

**Fig 5:**
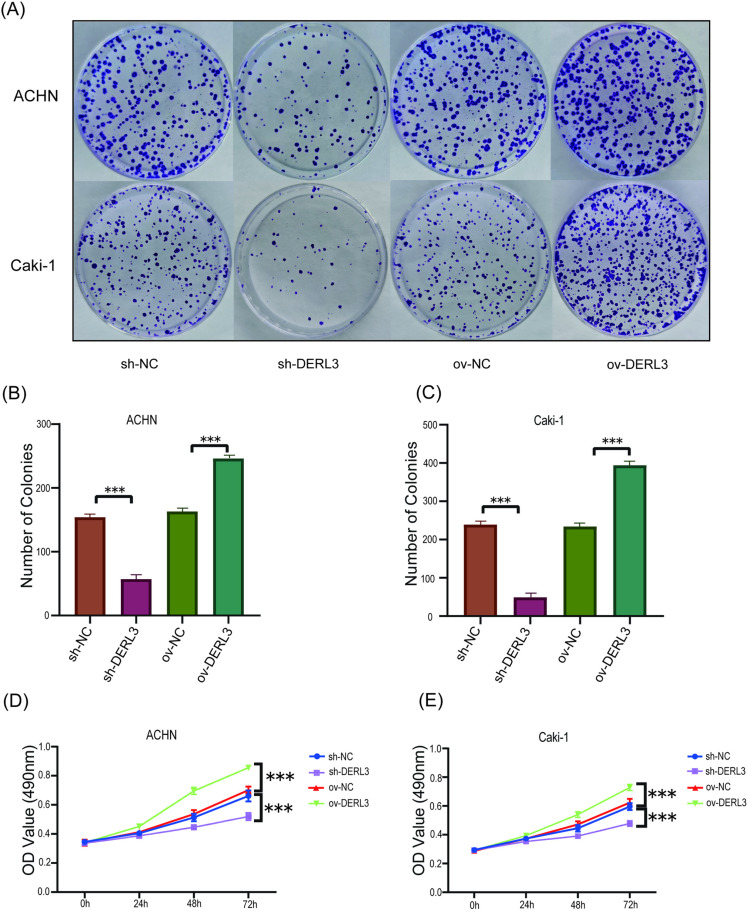
Effects of DERL3 overexpression or knockdown on proliferation of ccRCC cells. (A, B, C): Plate cloning experiments confirmed that overexpression of DERL3 significantly increased the number of ccRCC colonies, while knocking down DERL3 expression reduced the number of ccRCC colonies; (D, E): MTT experiments showed that the Optical Density in the DERL3 overexpression group was significantly increased, while that in the DERL3 knockdown group was significantly decreased; (***p<0.001).

**Fig 6:**
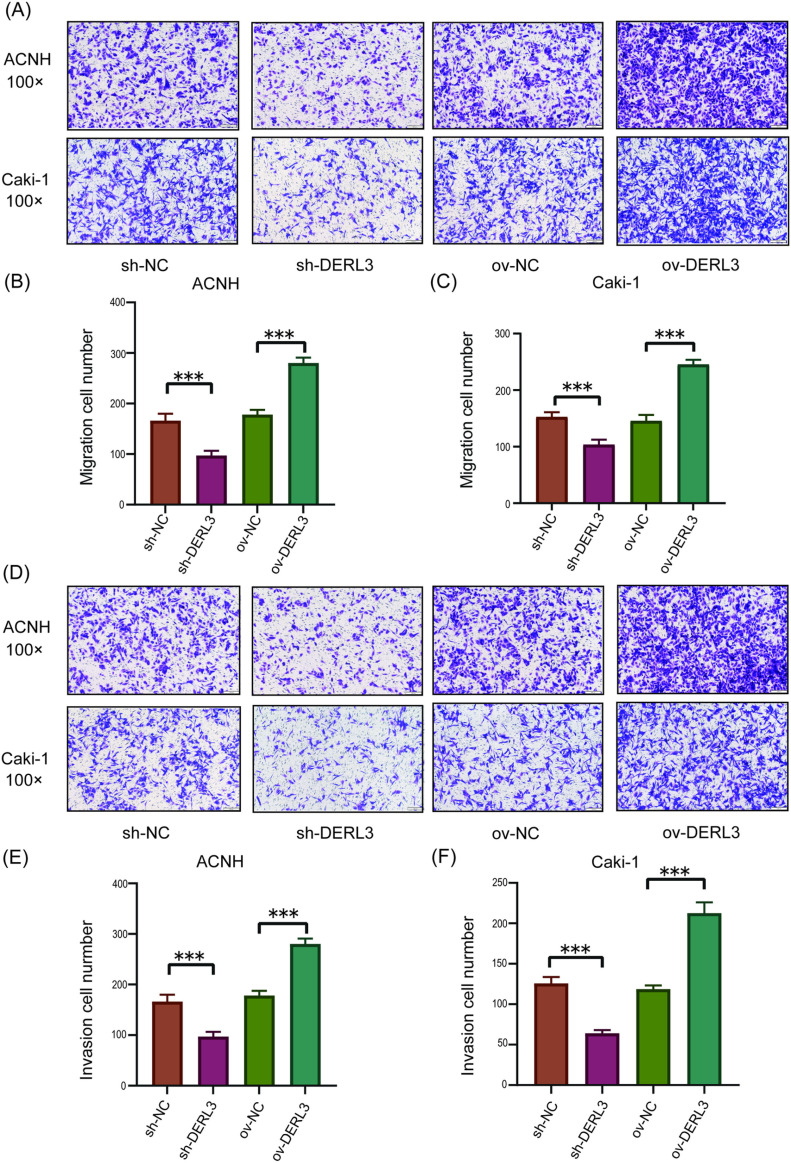
Effects of DERL3 overexpression or knockdown on ccRCC cell migration and invasion. (A, B, C): Transwell migration assay showed that DERL3 overexpression promoted ccRCC migration, while DERL3 knockdown inhibited ccRCC migration; (D, E, F): Transwell invasion assay showed that DERL3 overexpression enhanced ccRCC invasion, while DERL3 knockdown inhibited ccRCC invasion; (***p<0.001).

To investigate the influence of DERL3 expression on the growth and migratory capabilities of ccRCC, we conducted MTT and Transwell assays, in addition to assessing the proliferation capability of ccRCC cells via colony formation assays. The colony formation assay revealed that the number of colonies in the ov-DERL3 group was significantly greater than that in the control group, while the sh-DERL3 group exhibited a noticeable reduction in colony count (Fig 5A–C). The results from the MTT assay indicated that the proliferation ability of ACHN and Caki-1 ccRCC cells was significantly enhanced in the DERL3 overexpression group (ov−DERL3) compared to the control group, while the proliferation ability of ccRCC cells in the sh-DERL3 group was significantly reduced (Fig 5D, E). Similarly, the Transwell assay results demonstrated that the ov-DERL3 group showed significantly increased migratory (Fig 6A–C) and invasive (Fig 6D–F) abilities compared to the control group, whereas the sh-DERL3 group exhibited a marked decrease in both migration and invasion (Fig 6).

In summary, elevated DERL3 expression in ccRCC enhances the proliferation, invasion, and metastasis of ccRCC cells.

### DERL3 facilitates epithelial-mesenchymal transition in ccRCC through the activation of TGF-β1 signaling

**Fig 7:**
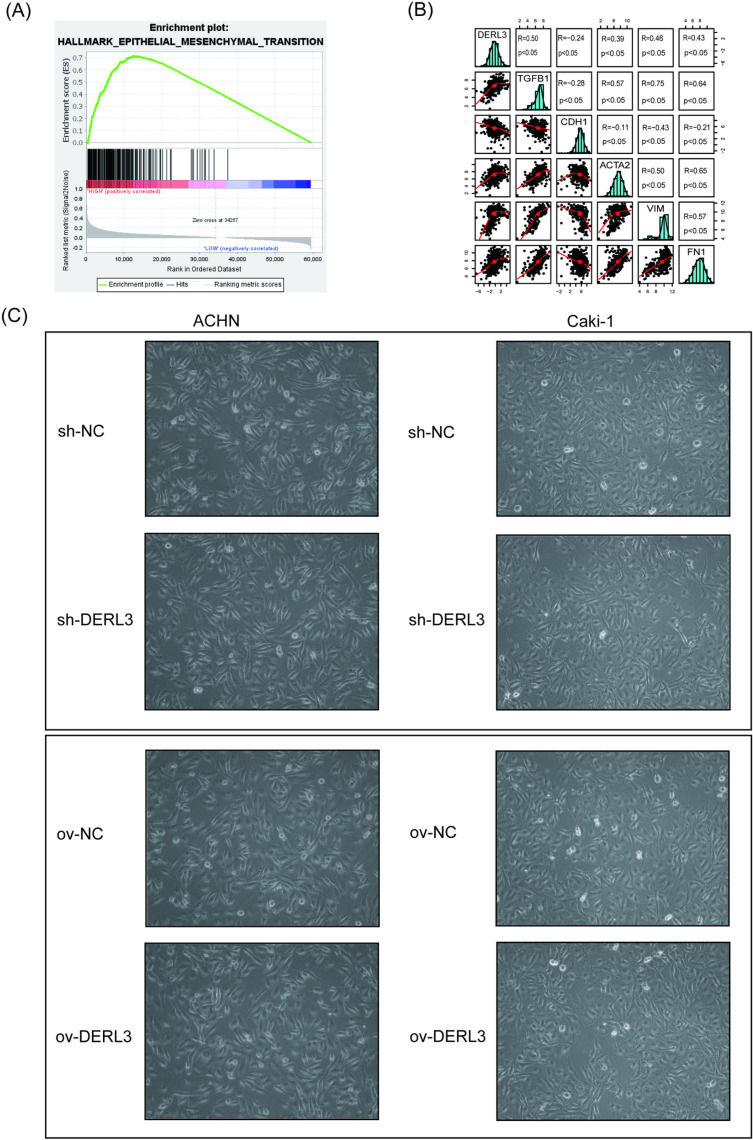
Gene set enrichment analysis (GSEA) and correlation analysis and phase contrast microscopy results based on TCGA database. (A): GSEA results show enriched pathways associated with high expression of DERL3; (B): Correlation analysis between DERL3 and TGFB1 as well as CDH1, ACTA2, VIM, and FN1; (C): Phase contrast microscopy observation of cell morphology DERL3 overexpressing cells mainly showed mesenchymal-like morphology, while DERL3 silenced cells showed more epithelial characteristics.

To further investigate the potential mechanisms by which high expression of DERL3 promotes the progression of ccRCC, we conducted an enrichment analysis using the GSEA method. The results indicated a significant enrichment of genes associated with EMT (Fig 7A). Subsequently, we utilized the R programming language to analyze the relationship between DERL3 and various genes related to EMT, specifically TGFB1, as well as epithelial cell-associated proteins encoded by CDH1 and mesenchymal cell-associated proteins encoded by ACTA2, VIM, and FN1. The findings revealed that DERL3 expression was positively correlated with TGFB1 and the mesenchymal-associated genes ACTA2, VIM, and FN1, while exhibiting a negative correlation with the epithelial-associated gene CDH1 (Fig 7B). Phase contrast microscopy observations showed that ccRCC cells in the DERL3 high expression group mainly exhibited a loosely arranged, irregularly shaped mesenchymal cell-like morphology, while DERL3 knockdown cells showed more tightly arranged, square-shaped epithelial cell characteristics (Fig 7C).

**Fig 8:**
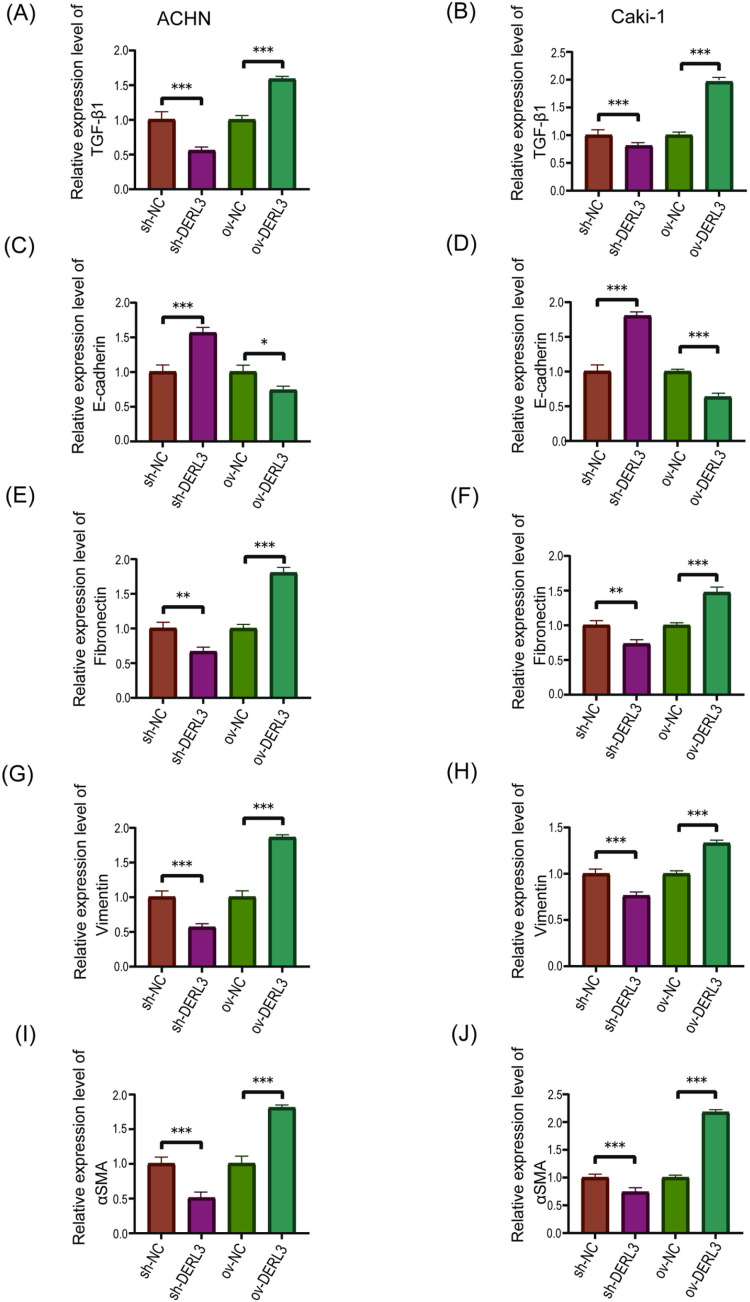
Gene expression detection of epithelial and stromal cell-related proteins in ccRCC cells after overexpression or knockdown of DERL3 expression. (A, B): The expression of TGF-β1 was increased in the DERL3 overexpression group, and decreased in the DERL3 knockdown group; (C, D): The mRNA expression of epithelial cell-associated protein E-cadherin was decreased in the DREL3 overexpression group and highly expressed in the DERL3 knockdown group; (E, F, G, H, I, J): The mRNA expression of interstitial cell-related proteins Fibronectin(E, F), Vimentin(G, H), and α-SMA(I, J) was increased in the DREL3 overexpression group and was low in the DERL3 knockdown group; (*p<0.05, **p<0.01, ***p<0.001).

**Fig 9:**
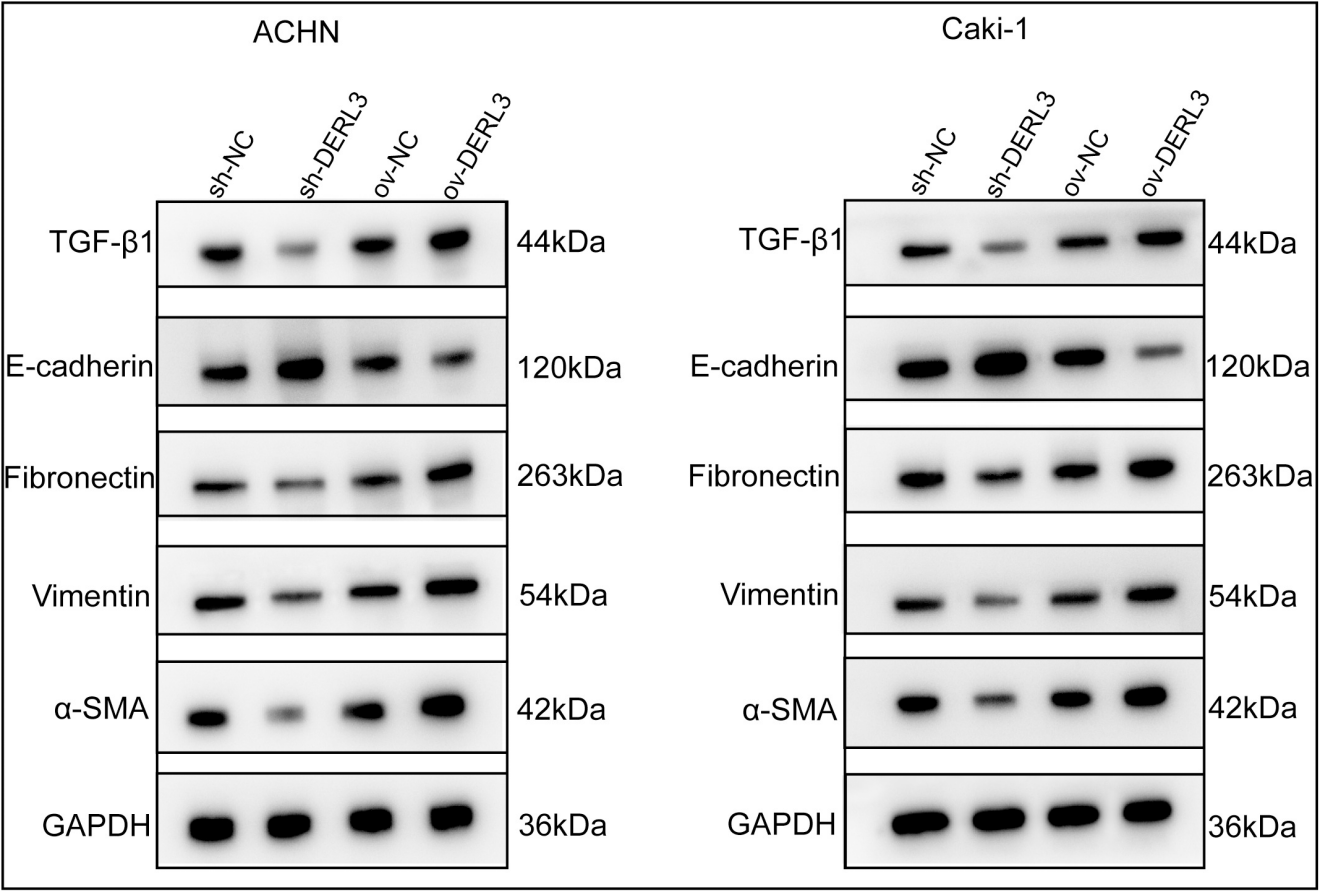
Western blot verification of the relationship between DERL3 expression and TGF-β1 and epithelial and mesenchymal cell-related proteins. In the DERL3 overexpression group, the expression of epithelial cell-related protein E-cadherin was decreased, and the expression of TGF-β1 protein and mesenchymal cell-related proteins Fibronectin, Vimentin, and α-SMA was increased in ccRCC cells. In the DERL3 knockdown group, the expression of epithelial cell-related protein E-cadherin was increased, and the expression of TGF-β1 protein and mesenchymal cell-related proteins Fibronectin, Vimentin, and α-SMA was decreased in ccRCC cells.

Furthermore, to elucidate the relationship between DERL3 and the EMT-related gene TGFB1, RT-qPCR analysis revealed a significant increase in TGF-β1 expression in the DERL3 high-expression group (ov−DERL3) compared to the control group (ov−NC) (Fig 8A, B). Conversely, the expression of TGF-β1 was significantly reduced in the DERL3 low-expression group (sh−DERL3) compared to the control group (sh−NC) (Fig 8A, B). Additionally, RT-qPCR results demonstrated a decrease in the expression of the epithelial-associated protein E-cadherin in the DERL3 high-expression group (ov−DERL3) compared to the control group (ov−NC) (Fig 8C, D), while there was an increase in the expression of mesenchymal-associated proteins Fibronectin (Fig 8E, F), Vimentin (Fig 8G, H), and α-SMA (Fig 8I, J). Conversely, in the DERL3 low-expression group sh−DERL3 compared to the control group sh−NC, the expression of E-cadherin increased (Fig 8C, D), while the expression of Fibronectin (Fig 8E, F), Vimentin (Fig 8G, H), and α-SMA (Fig 8I, J) decreased. Our Western Blot experiments corroborated the RT-qPCR results (Fig 9).

**Fig 10:**
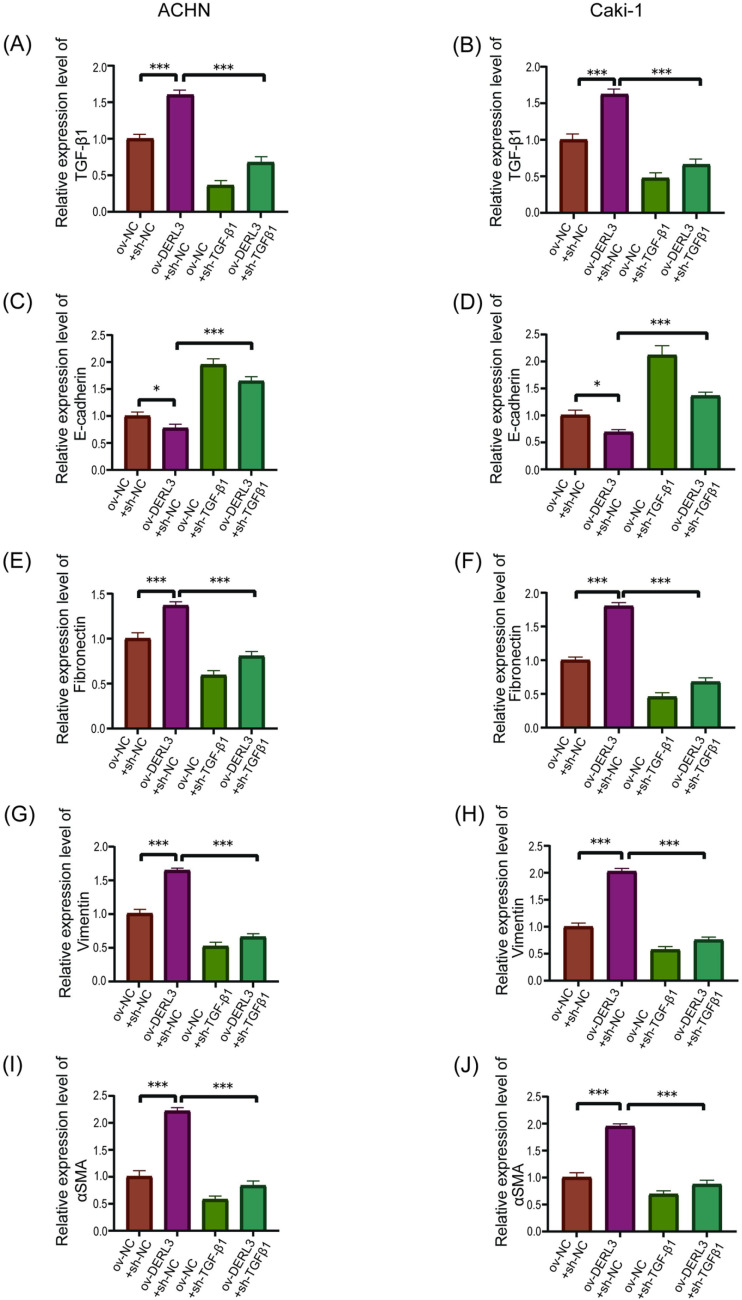
qRT-PCR validation of the relationship between DERL3 expression and TGF-β1 and epithelial-mesenchymal cell-related proteins. After overexpression of DERL3 in ccRCC cells and subsequent knockdown of TGF-β1 (A, B), the mRNA expression of the epithelial cell-related protein E-cadherin (C, D) increased, while the mRNA expression of the mesenchymal cell-related proteins Fibronectin (E, F), Vimentin (G, H), and α-SMA (I, J)decreased; (*p<0.05, **p<0.01, ***p<0.001).

**Fig 11:**
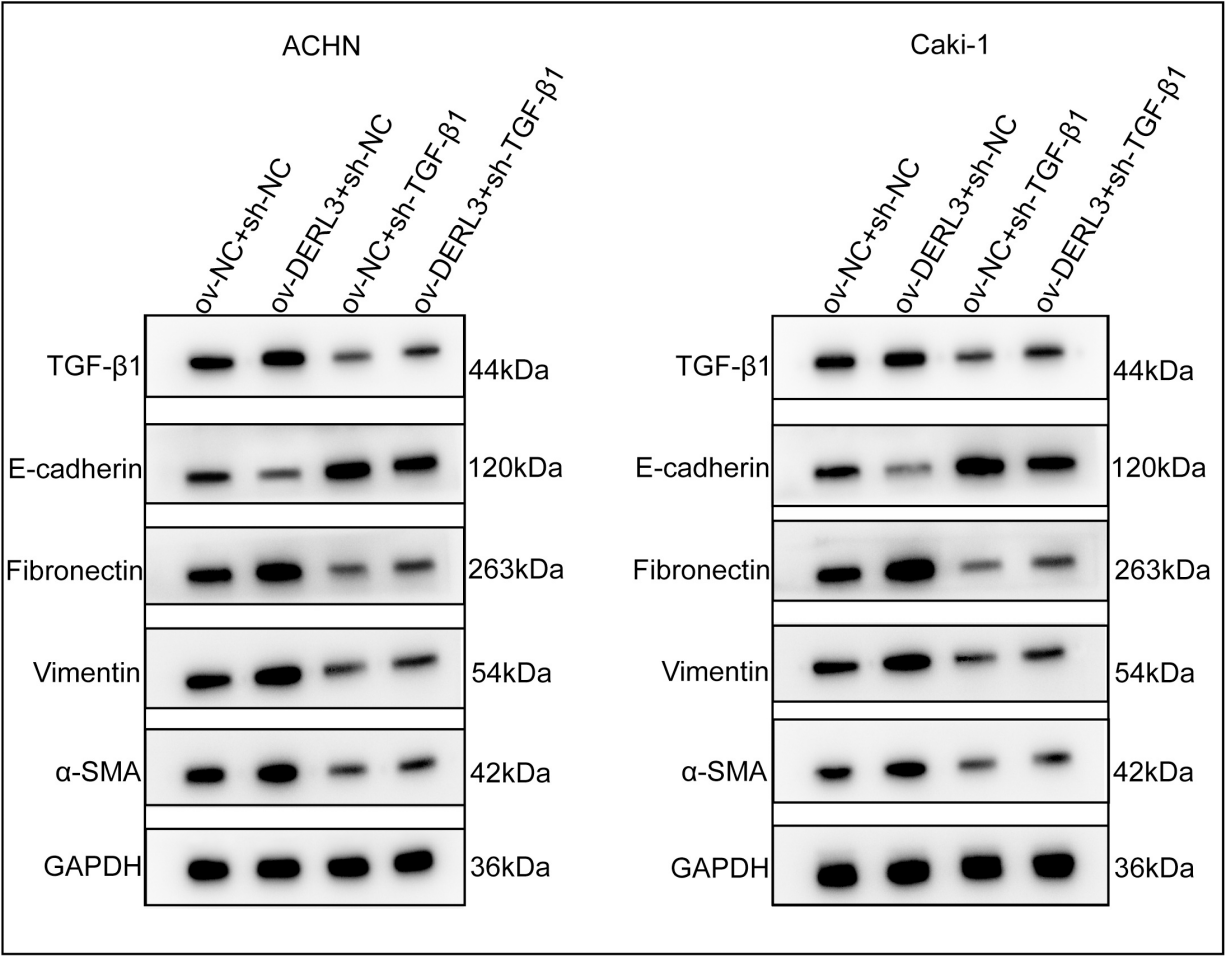
Western blot verifies the relationship between DERL3, TGF-β1 and epithelial-mesenchymal transition. After overexpression of DERL3 in ccRCC cells and then knockdown of TGF-β1, the expression of epithelial cell-related protein E-cadherin increased, and the expression of mesenchymal cell-related proteins Fibronectin, Vimentin, and α-SMA decreased.

To further confirm that the overexpression of DERL3 promotes EMT through TGFB1, we conducted RT-qPCR to examine the expression levels of epithelial and mesenchymal cell-associated protein genes following the knockdown of TGFB1 in DERL3-overexpressing cells (Fig 10A, B). The results indicated that, compared to the control group, overexpression of DERL3 led to a decrease in the expression of the epithelial cell-related protein gene E-cadherin (Fig 10C, D). Simultaneously, the expression levels of mesenchymal cell-related protein genes, including Fibronectin (Fig 10E, F), Vimentin ((Fig 10G, H), and α-SMA ((Fig 10I, J), increased. However, upon knockdown of TGF-β1 (Fig 10A, B), the expression of E-cadherin increased (Fig 10C, D), while the levels of Fibronectin ((Fig 10E, F), Vimentin ((Fig 10G, H), and α-SMA ((Fig 10I, J) decreased. Additionally, Western blot analysis was performed to assess the expression of epithelial and mesenchymal cell-associated proteins in DERL3-overexpressing cells after TGF-β1 knockdown. The findings revealed that, in comparison to the control group, there was a reduction in E-cadherin expression following DERL3 overexpression, accompanied by increased expression of Fibronectin, Vimentin, and α-SMA. Conversely, upon TGF-β1 knockdown, E-cadherin expression increased while Fibronectin, Vimentin, and α-SMA expression levels decreased (Fig 11).

## Discussion

Renal cell carcinoma is one of the most prevalent malignant tumors of the urinary system, with approximately one-third of patients exhibiting metastasis at the time of initial diagnosis. Even among those receiving surgical treatment in the early stages, nearly 30% experience recurrence or metastasis, which may ultimately lead to mortality [1]. Changes in the von Hippel-Lindau VHL tumor suppressor gene, located on the short arm of chromosome 3, have been identified as significant contributors to the development of ccRCC [22]. Additionally, high-frequency mutations in chromatin modification genes, such as PBRM1, SETD2, BAP1, ARID1A, and MLL3, have also been documented [22,23]. The treatment landscape for metastatic RCC has evolved from initial systemic chemotherapy to cytokine therapy, followed by targeted therapy and immune checkpoint inhibitor therapy. Currently, the management of metastatic RCC primarily relies on a combination of immunotherapy and targeted therapy.

Hypoxia, malnutrition, the accumulation of intracellular reactive oxygen species, abnormal pH, and inflammation can lead to aberrant intracellular protein folding and result in endoplasmic reticulum stress [24]. Derlin-3, a significant member of the Derlin family, plays a pivotal role in the ER stress process. Specifically, it is involved in the formation of ERAD complexes during episodes of ER stress. Furthermore, research indicates that Derlin-3, as an ER transmembrane protein, is implicated in the ubiquitination of misfolded proteins [10]. Peter J. Belmont’s research highlights that the overexpression of DERL3 can protect myocardial cells from ischemic injury, whereas decreased expression of DERL3 enhances ischemia-induced cardiac cell death [25]. Current research indicates that Derlin-3 plays a role in the development of various cancers, including breast and lung cancer [12,15,26,27]. Additionally, studies suggest that endoplasmic reticulum stress is implicated in the progression of ccRCC, with the expression of DERL1, DERL3, and other members of the endoplasmic reticulum stress-related protein family being associated with the epithelial-mesenchymal transition in lung and bladder cancers [28–30]. However, there are currently no reports addressing the role of DERL3 in renal cancer.

This study initially identified that DERL3 is highly expressed in ccRCC through a comprehensive analysis of multiple databases. Further examination of RNA-seq data from public databases revealed a significant correlation between DERL3 expression and the prognosis of patients with ccRCC. Additionally, we utilized qRT−PCR and Western blotting to confirm that DERL3 exhibits elevated expression in ccRCC cell lines, particularly in the ACHN and Caki-1 metastatic cell lines. IHC performed on clinical samples of ccRCC and adjacent non-cancerous tissues demonstrated that the expression of DERL3 was significantly higher in ccRCC tissues compared to paracancerous tissues. Furthermore, GSEA indicated that the epithelial-mesenchymal transition pathway was significantly enriched.

TGF-β1 has been implicated in the EMT of various tumors [28,31–35]. Our analysis of data from TCGA revealed a positive correlation between the expression of TGFB1 and DERL3. This finding was further validated through RT-qPCR and Western blot analyses. Additionally, we knocked down the expression of TGFB1 in cells with elevated DERL3 levels, observing an increase in epithelial cell adhesion protein E−cadherin expression and a decrease in the expression of mesenchymal cell-related proteins, including Fibronectin, Vimentin, and α-SMA. These results suggest that high DERL3 expression may facilitate the EMT of ccRCC through the action of TGF-β1, thereby promoting the progression of ccRCC.

While our study enhances the treatment strategies for ccRCC, we acknowledge its limitations. The transcriptomic data analyzed in this study were derived from databases primarily composed of European and American populations. Due to racial differences, the findings may have certain limitations in their applicability to Asian populations. Future studies should include more data from Asian populations to validate the cross-racial applicability of these results. In the experimental validation of DERL3’s role in promoting the EMT in renal cancer, we initially utilized only one epithelial cell marker protein. We plan to further validate additional epithelial cell marker proteins in subsequent studies to more comprehensively elucidate the role of DERL3 in EMT. The regulatory mechanisms by which DERL3 influences TGFB1 expression and other signaling pathways remain inadequately understood. Based on existing literature, we speculate that DERL3 may influence the stability or secretion of TGFB1 protein through its role in endoplasmic reticulum-associated degradation (ERAD) or other post-translational regulatory pathways. Further investigation and clinical validation of these intricate molecular processes are essential for advancing targeted therapies for ccRCC.

## Conclusion

In summary, this study confirmed that DERL3 is overexpressed in ccRCC and that its elevated expression is associated with poor prognosis. The mechanism underlying this association involves the promotion of the epithelial-mesenchymal transition process of ccRCC through the regulation of TGFB1 expression. Consequently, DERL3 has the potential to serve as a novel target for the clinical treatment of ccRCC.

## Consent for publication

All patients or their caregivers signed a consent form giving permission to use their anonymous data for research.

## Supporting information

S1 Fig Western blot original image. (PDF)

S2 Fig Quantitative analysis by western blot (Fig 9). (TIF)

S3 Fig Quantitative analysis by western blot (Fig 11). (TIF)

S4 File Code. (ZIP)
