# Supplementary material for: DERL3 facilitates the progression of clear cell renal cell carcinoma by promoting epithelial-mesenchymal transition via regulation of the TGFB1 pathway
Source: PLoS One. 2025 Apr 29;20(4):e0322172. doi: 10.1371/journal.pone.0322172 (PMC12040103; doi:10.1371/journal.pone.0322172)
Supplement: S1 Fig — (PDF) [file pone.0322172.s001.pdf]

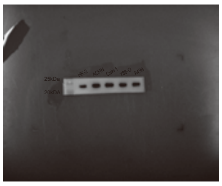

DERL3, 25kDa
Fig. 1G

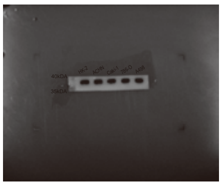

GAPDH, 36kDa
Fig. 1G

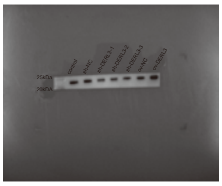

DERL3, 25kDa
Fig. 3C

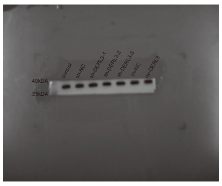

GAPDH, 36kDa
Fig. 3C

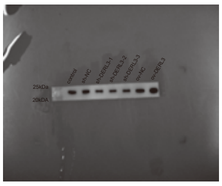

DERL3, 25kDa
Fig. 3D

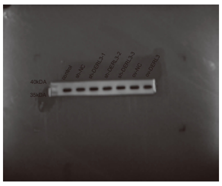

GAPDH, 36kDa
Fig. 3D

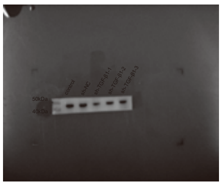

TGF-β1, 44kDa
Fig. 4C

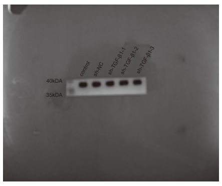

GAPDH, 36kDa
Fig. 4C

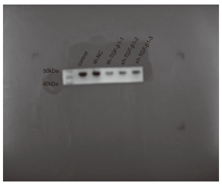

TGF-β1, 44kDa
Fig. 4D

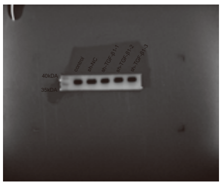

GAPDH, 36kDa
Fig. 4D

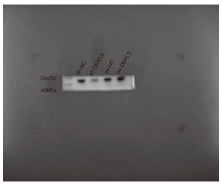

TGF-β1, 44kDa
Fig. 9 ACHN

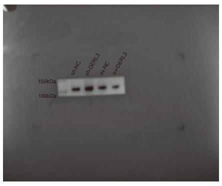

E-cadherin, 120kDa
Fig. 9 ACHN

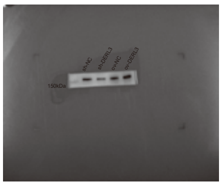

Fibronectin, 263kDa
Fig. 9 ACHN

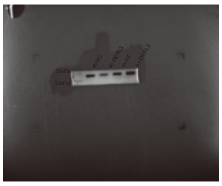

Vimentin, 54kDa
Fig. 9 ACHN

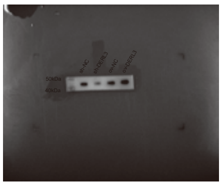

α-SMA, 42kDa
Fig. 9 ACHN

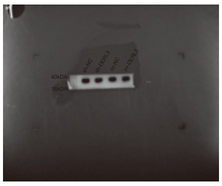

GAPDH, 36kDa
Fig. 9 ACHN

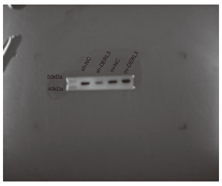

TGF-β1, 44kDa
Fig. 9 Caki-1

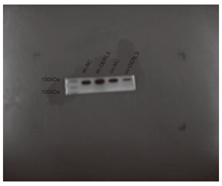

E-cadherin, 120kDa
Fig. 9 Caki-1

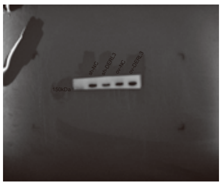

Fibronectin, 263kDa
Fig. 9 Caki-1

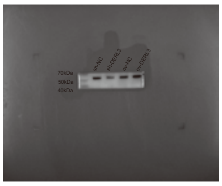

Vimentin, 54kDa
Fig. 9 Caki-1

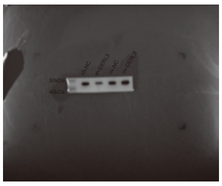

α-SMA, 42kDa
Fig. 9 Caki-1

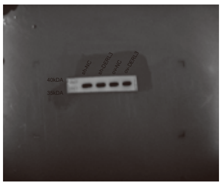

GAPDH, 36kDa
Fig. 9 Caki-1

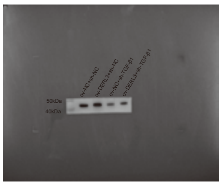

TGF-β1, 44kDa
Fig. 11 ACHN

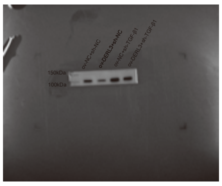

E-cadherin, 120kDa
Fig. 11 ACHN

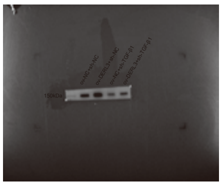

Fibronectin, 263kDa
Fig. 11 ACHN

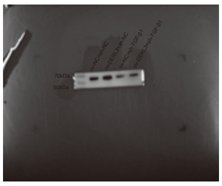

Vimentin, 54kDa
Fig. 11 ACHN

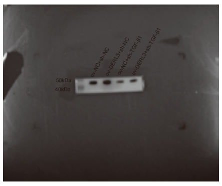

α-SMA, 42kDa
Fig. 11 ACHN

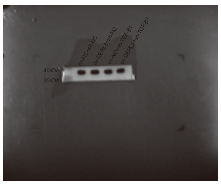

GAPDH, 36kDa
Fig. 11 ACHN

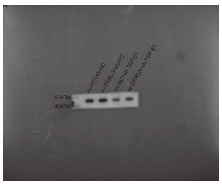

TGF-β1, 44kDa
Fig. 11 Caki-1

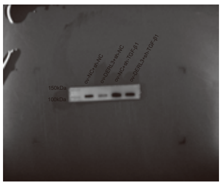

E-cadherin, 120kDa
Fig. 11 Caki-1

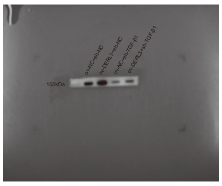

Fibronectin, 263kDa
Fig. 11 Caki-1

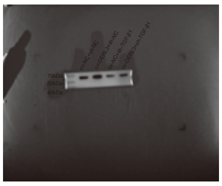

Vimentin, 54kDa
Fig. 11 Caki-1

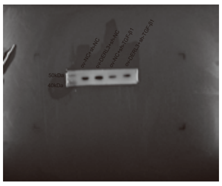

α-SMA, 42kDa
Fig. 11 Caki-1

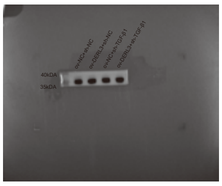

GAPDH, 36kDa
Fig. 11 Caki-1
