# Supplementary figures and images for: DERL3 facilitates the progression of clear cell renal cell carcinoma by promoting epithelial-mesenchymal transition via regulation of the TGFB1 pathway
Source: PLoS One. 2025 Apr 29;20(4):e0322172. doi: 10.1371/journal.pone.0322172 (PMC12040103; doi:10.1371/journal.pone.0322172)

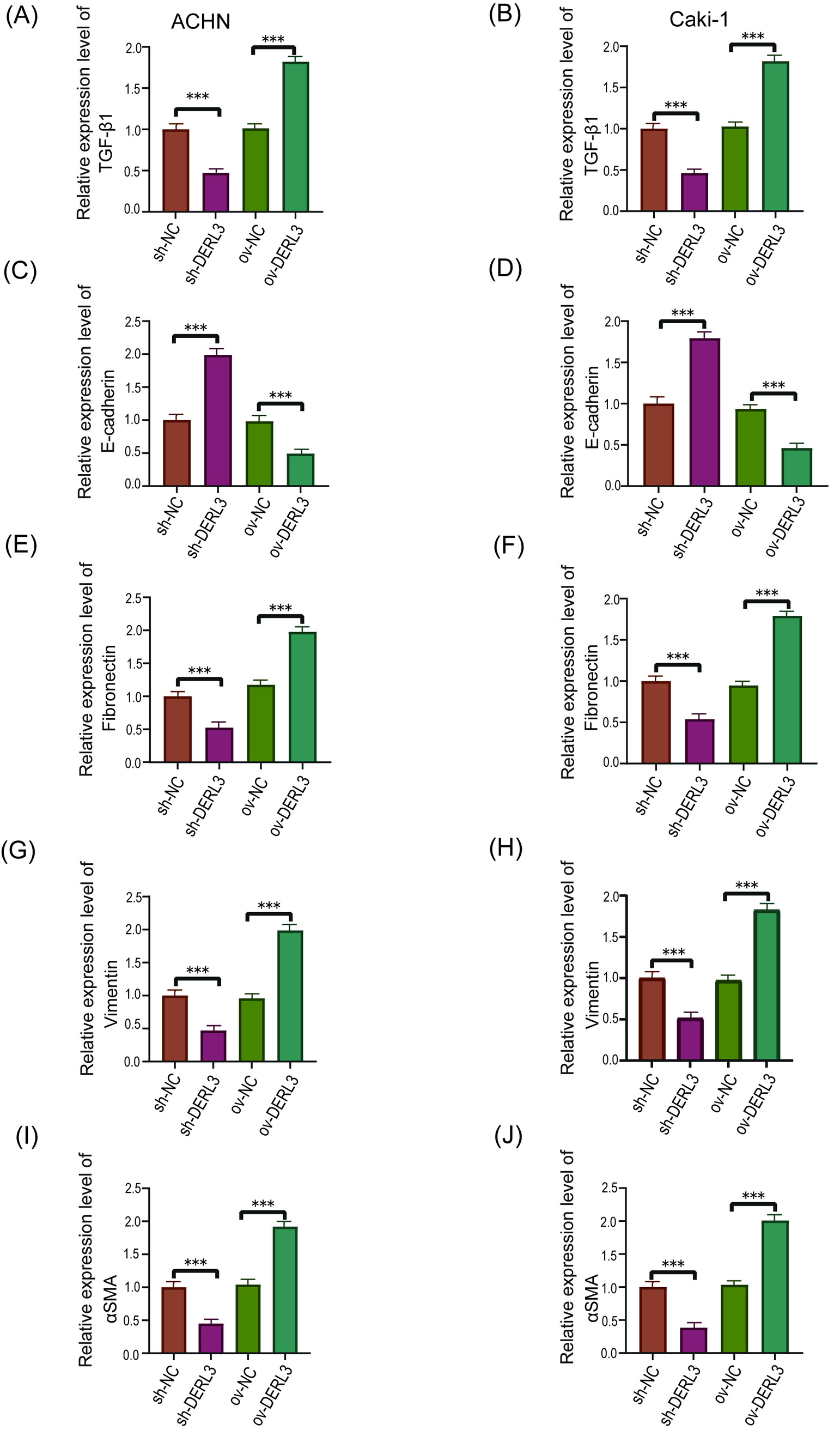

Supplement: S2 Fig — (TIF) [file pone.0322172.s002.tif]

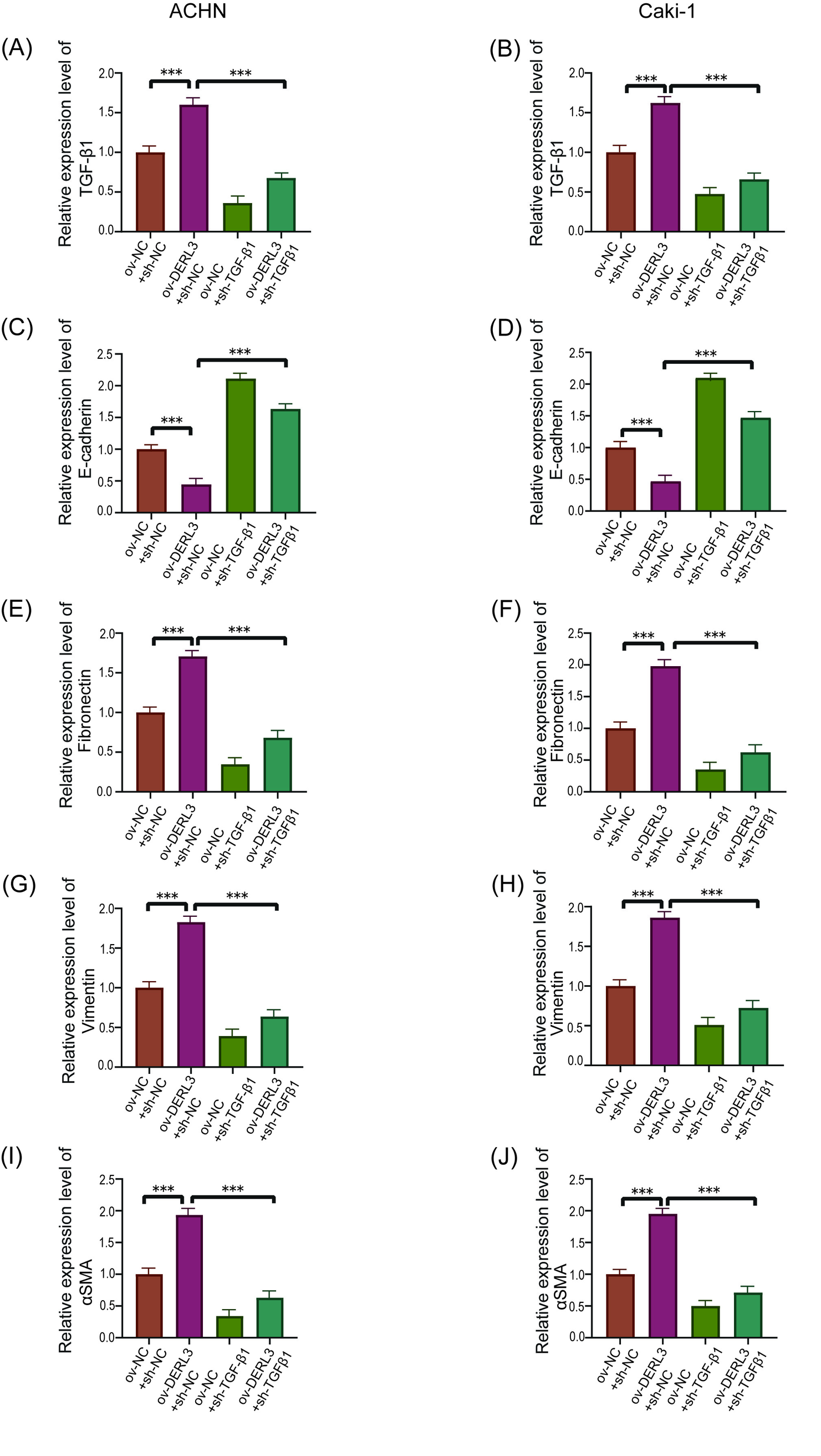

Supplement: S3 Fig — (TIF) [file pone.0322172.s003.tif]
